# Cameroon public health sector: shortage and inequalities in geographic distribution of health personnel

**DOI:** 10.1186/s12939-015-0172-0

**Published:** 2015-05-12

**Authors:** Tinyami Erick Tandi, YongMin Cho, Aba Jean-Cluade Akam, Chick Ofilia Afoh, Seung Hun Ryu, Min Seok Choi, KyungHee Kim, Jae Wook Choi

**Affiliations:** Institute for Occupational and Environmental Health, Korea University, Seoul, South Korea; Ministry of Public Health Yaounde, Yaounde, Cameroon; Department of Preventive Medicine, School of Medicine, Graduate School of Public Health, Korea University, Seoul, South Korea

**Keywords:** Health workforce, Availability, Distribution, Inequalities, Alternative indicators, Cameroon

## Abstract

**Introduction:**

Cameroon is classified by the World Health Organization (WHO) as having a critical shortage of health personnel. This is further complicated by the geographic distributional inequalities of the national health workforce. This shortfall impedes Cameroons’ progress of improving the human resources for health (HRH) to meet up with the Millennium Development Goals (MDGs) by 2015. However, it is unknown whether the health workforce of Cameroon is distributed equally across geographic regions. Additionally, indicators other than population levels have not been used to measure health care needs. This study aimed to assess the adequacy, evenness of distribution and challenges faced by the health workforce across the different regions of Cameroon.

**Methods:**

National health personnel availability and distribution were assessed by use of end-of-year census data for 2011 obtained from the MoPH data base. The inequalities and distribution of the workforce were estimated using Gini coefficient and Lorenz curve and linear regression was used to determine the relation between health personnel density and selected health outcomes. Alternative indicators to determine health care needs were illustrated using concentration curves.

**Results:**

Significant geographic inequalities in the availability of health workforce exist in Cameroon. Some regions have a higher number of physicians (per person) than others leading to poor health outcomes across the regions. 70 % of regions have a density of health personnel-to-population per 1,000 that is less than 1.5, implying acute shortage of health personnel. Poor working and living conditions, coupled with limited opportunities for career progress accounted for some documented 232 physicians and 205 nurses that migrated from the public sector. Significant distributional inequality was noticed when under-five infant mortality and malaria prevalence rate were used as indicators to measure health care needs.

**Conclusion:**

Our results show an absolute shortage of public health personnel in Cameroon that is further complicated by the geographic distributional inequalities across the regions of the nation. Cameroon aims to achieve universal health coverage by 2035; to realize this objective, policies targeting training, recruitment, retention and effective deployment of motivated and supported health workforce as well as the development and improvement of health infrastructures remain the major challenge.

## Introduction

The public health sector is considered one of the driving forces of most developing countries’ health systems, due to some of its core objectives of preventing, improving and providing health services to their populations [1]. Although the World Health Organization (WHO) defines human resources for health (HRH) as “all people engaged in actions whose primary intent is to enhance health” [1], in this study we restricted our findings to health personnel of the public health sector. The estimated global shortage of HRH is above 4 million, assuming all countries have to attain an average worker-to-population density of 2.5 per 1,000 [2]. According to the 2006 World Health Report, the WHO estimated that over 4.3 million more health workers are needed to bridge the gap of health personnel globally, of which about 1.5 million (35 %) are required in Africa alone [1].

Across the world, 57 countries have been identified as having “critical shortages” of health workers, amongst which 36 are in Africa including Cameroon [3]. However, for many low and middle-income countries to attain specific Millennium Development Goals (MDGs) pertaining to health, they need to reduce the shortages of trained, motivated and supported health workforce, as health personnel have a direct/indirect role in strengthening societal health that is strongly linked with sustainability of human and economic development [4]. This is more critical in the Sub-Saharan Africa (SSA), where the existing low densities in health workforce to population per 1,000, have further strained their ability to cope with increasing health crisis and disease burden as compared to other regions in the world [5]. In addition to the low availability of health personnel in the SSA where Cameroon is found, there is a common understanding that the region is further exacerbated by geographic distributional inequalities that is even more severe in rural areas [5]. However, one of the major constraints to this postulate has been limited in-country data on their HRH profiles, as most studies have focused themselves to the distribution of specific health cadres (physicians or nurses), [6–9].

The pro-urban uneven distribution of the available health workforce due to poor social amenities in the rural areas is of major concern, for example, in the city of Nairobi in Kenya, a physician catered to 500 persons, while a physician in the Turkana district catered to 160,000 persons within the same country [10]. Similarly, in Cameroon, a physician in the Center region catered to 5,449 persons as opposed to 26,726 persons to a physician in the Adamawa region [11]. In this article, we use the 2011 HRH census in Cameroon to analyze health personnel distributional patterns across the different regions of the country.

Cameroon is located in Central Africa with a population of 20,549,221 [12]. It is partitioned into ten administrative regions which are further divided into 58 districts, 360 sub-districts and 339 councils [13].

The public health sector of the country is pyramidal, and has a centralized system of administration that runs from the central (ministry), through the intermediary (regional delegations), and cumulating at the peripheral (health districts) levels. Three different levels of health care delivery services exist in Cameroon; the tertiary, the secondary, and the primary services. However, intra-regional differences in health personnel availability which may be associated with urban/rural divide and corresponding economic disparities, could not be assessed due to lack of district-level data. Our study was therefore limited to inter-regional inequalities in the availability of the public health sector health workforce in 2011.

Distributional inequalities of health personnel are often assessed by comparing the number of available health personnel across different geographic regions [14, 15]. One of the objectives of this paper is to provide a quantitative description of distributional inequalities of health personnel across the different regions in Cameroon. In this way, we are able to assess if regions that have relatively few physicians are compensated by having more lower health cadres (nurses and paramedical).

Just as death rate is used as an indicator in high-income countries [16, 17], indicators such as under-five mortality and malaria prevalence can be used in low-income countries like Cameroon. In this study we use under-five infant mortality and malaria prevalence rate as alternative indicators to population levels in the partial determination of health care needs in Cameroon as malaria accounts for more than 40 % of deaths in the nation.

Although, these indicators may present a partial measurement of the healthcare needs in Cameroon, they definitely give a reflection of the possible regional differences in health care needs when evaluating the adequacy of health personnel across the different regions of the country. It is useful that under-five infant mortality is an important tool in determining health care needs in low-income countries where their annual mortality measures up to 30 % as opposed to less than 1 % in high income countries [14]. In Cameroon under-five mortality varies by a factor of three, ranging from 251 deaths in the Extreme north region to 112 in the Center region per 1,000 live births [18]. Also in 2010, malaria prevalence is about 36 % among Cameroonians [18–20]. Based on this, it will be of reasonable importance to use both indicators as a partial measurement of health care needs.

In Cameroon, HRH has suffered a great challenge as a result of the economic crisis that caused the Government to set in reforms in accordance with the Structural Adjustment Program (SAP) enacted by the World Bank and the International Monetary Fund (IMF) in the 1980s and 1990s [21, 22]. These reforms led to the suspension of recruitment of public health personnel, no development in health infrastructures as well as salary cuts for health personnel. This may have accounted for the poor working and living conditions of health workers ranging from low salary, overwork burden, no professional autonomy, limited opportunities for career progress and lack of sufficient as well as modern equipment for smooth and effective practices.

Although, very little has been analyzed on HRH in Cameroon, we performed this study to assess the adequacy, evenness of distribution and challenges faced by the health workforce across the different regions of the nation. Our findings are aimed towards informing public health policy makers interested in HRH to include further interventions in their planning agenda for the improvement and prevention of human resource crisis within the health system. In this study we refer to physicians, nurses and paramedics as the cadres involved with the provision of clinical health care services to the public.

## Methods

We extracted indicators (age, cadres, sex, location, population and facilities) from HRH indicator compendium that have been pre-tested and used for research [23] as a guide to our study. We obtained data regarding public health HRH from the MoPH database on the last census performed in 2011, aimed at taking stock of all health personnel of the nation. This database contains staff demographic particulars as well as health facilities in line with our indicators [24]. Population data, under-five infant mortality, maternal mortality, measles immunization coverage and malaria prevalence rate were obtained from the National Institute of Statistics Cameroon [18], and the Central Bureau of Census and Population Studies Cameroon (BUCREP), [13]. We also use some published literature available at the MoPH archive that addressed the working and living conditions of health personnel as well as the suspension in the development of health infrastructures in Cameroon. We use this information to analyze some of the challenges facing the health workforce of the nation.

### Ethical consideration

This study did not involve with the collection of primary data, therefore ethical clearance was not needed for the study. However, permission was obtained from the Ministry of Public health, department of human resource for health to access the information used.

### Data analysis

Data analysis was done by the use of descriptive statistical method to create tables that grouped and classified the different cadres, ages, sex, and the geographical distribution of the health personnel and population across the regions of the country. The relation between selected health personnel densities and outcomes was assessed by linear regression of data on maternal mortality, under-five infant mortality and measles immunization coverage of the nation in 2011 [24] using IBM SPSS version 21.0 software package.

**Fig. 1:**
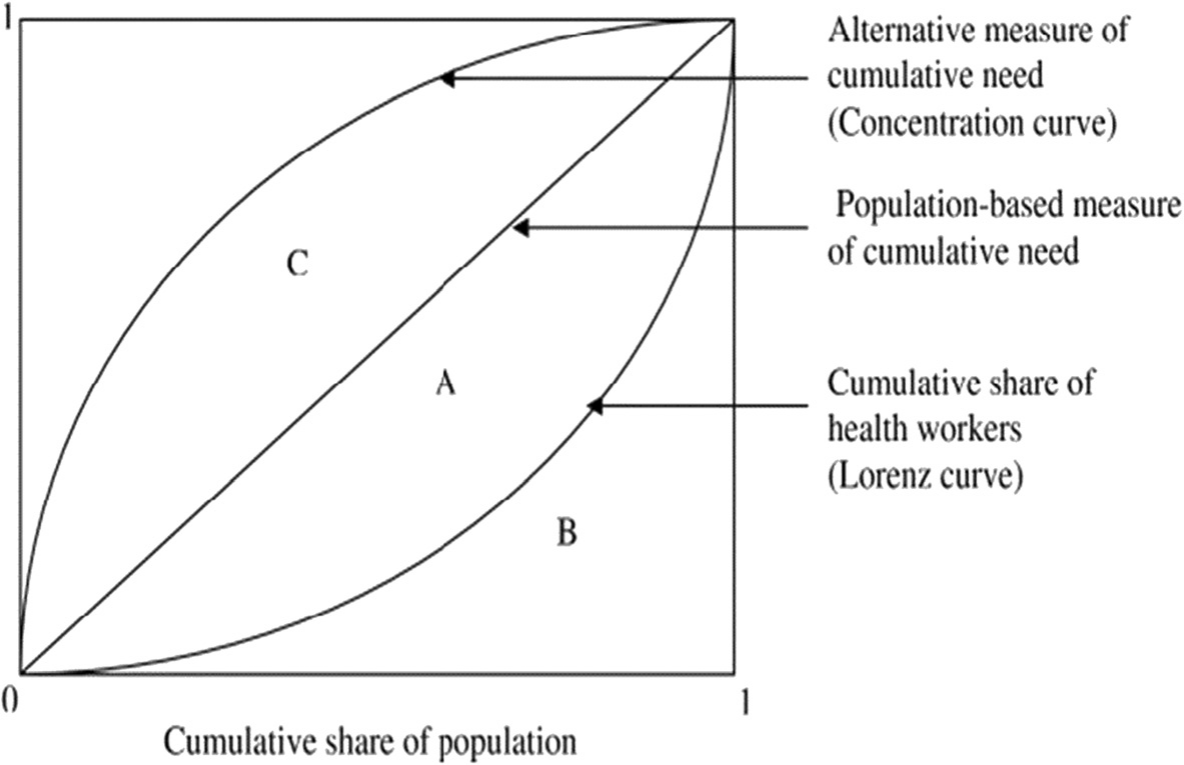
Illustration of Lorenz curve and concentration curve

The national health workforce distributional inequality was measured using Gini coefficient and Lorenz curve. Lorenz curves was used to characterize the distribution of health personnel and show the cumulative share of health workers against cumulative population share when the different locations are ranked from the lowest to the highest number of health personnel. The Gini index was used to measure the aggregate level of inequality with values ranging between 0 and 1, with higher values indicating higher levels of inequality. This was calculated as;

$$ G=\frac{n+1}{n}-\frac{2{\displaystyle {\sum}_1^n\left(n+1-i\right){x}_i}}{n{\displaystyle {\sum}_1^n{x}_i}}, $$

where G is the Gini index, n is the number of observations and X_*i*_ the number of health personnel at *i*th location. Gini coefficient and Lorenz curve have been use in previous studies to measure health personnel inequalities [12, 14]. Also, we use concentration curves which have been used to typify socioeconomic inequalities in health [22, 25, 26], to describe the alternative ways of determining health care needs using indicators such as under-five infant mortality and malaria prevalence rate. Using the concentration curves, we graphically illustrate on the same diagram as the Lorenz curve, the importance of using alternative indicators of health care needs to show how the equitable distribution of health personnel could be determine using alternative measures of need. In order to plot our concentration curves, we calculated the concentration index which is done similarly as the Gini index. Figure 1, shows a graphical annotation of Gini index (A/ (A + B) and concentration index C/ (A + B). When the concentration curve lies above or below the diagonal line, the region “C” is given a negative or a positive value respectively.

Congruent to the Gini index, the concentration index takes values from −1 and + 1, where index “0” means that the alternative measure of needs does not influence the overall level of inequality relative to when need is determine using the number of inhabitants. With a negative index value in which case the concentration curve lies above the diagonal, the demand for health care needs are higher in regions with proportional fewer health personnel. This implies that inequalities are larger when alternative measures of health care needs are used. Contrarily, inequalities will be smaller when the concentration curve lies below the diagonal with a positive concentration index implying health care needs are on an average proportionally distributed with the health workforce.

## Results

### Health personnel density, inequality and measure of alternative need

Our results provide a snapshot of the 2011 HRH situation of the public health sector in Cameroon. With regards to nationwide health workforce density representation, the aggregate density of health personnel-to-population was 1.3 per 1,000, which is lower than the WHO’s recommended critical shortage threshold of 2.5 [2]. There is a four-fold variation in the density of available health personnel-to-population per 1,000 from 2.0 to a low of 0.2 across the region as shown on Table 1. In Cameroon, none of the regions was able to attain the WHO critical shortage threshold limit, six out of the ten regions had densities below 2.0 and 3 regions below 1.0 and only 1 region (Center) with 2.0. Also, the ratio of physicians to nurses varies from 1:5 in the Center region to 1:21.5 in the North region. Among the different health personnel cadres to population per 1,000, the densities vary from 0.67 nurses per 1,000 to a low of 0.0 pharmacists per 1,000 as illustrated on Table 1. Table 2 presents the regression results of selected national health outcomes and specific health personnel density. The results depicts that the degree of association between the densities of physicians, nurses and paramedics varies across the different regions of the nation when maternal mortality, under 5 years infant mortality and measles immunization coverage were used to measure health outcomes. Measles immunization coverage showed significant coefficients with *p*-value of <0.001 as compared to under-five infant and maternal mortality. Therefore a change in the density of physicians, nurses and paramedics will influence the coverage of measles immunization and the outcomes of maternal and under 5 years infant mortality.

**Table 1:** The density of health personnel-to-population per 1,000, per region and Gini index per cadre.2011

Occupation regions	General public health personnel	Physicians	Nurses	Paramedics	Pharmacist	Administration	Social assistant agents	Community pharmacist agents	Support staff	Ratio of nurses per physician per region
Adamawa	0.84	0.04	0.59	0.12	0.00	0.05	0.00	0.00	0.05	15.7
Centre	1.97	0.18	0.92	0.27	0.01	0.19	0.02	0.02	0.35	5.00
East	1.35	0.06	0.85	0.20	0.00	0.05	0.03	0.04	0.11	14.50
Extreme North	0.78	0.02	0.40	0.08	0.00	0.01	0.04	0.04	0.18	20.00
Littoral	1.29	0.10	0.66	0.17	0.00	0.04	0.00	0.04	0.28	6.400
North	0.50	0.01	0.31	0.05	0.00	0.02	0.00	0.03	0.07	21.50
North West	1.05	0.04	0.50	0.12	0.00	0.06	0.03	0.07	0.22	12.30
West	1.72	0.05	0.97	0.21	0.00	0.04	0.06	0.11	0.29	19.00
South	1.36	0.08	0.80	0.21	0.00	0.09	0.02	0.03	0.14	10.60
South West	1.69	0.05	0.87	0.17	0.01	0.05	0.01	0.07	0.46	16.00
Total	1.29	0.07	0.67	0.16	0.00	0.06	0.02	0.05	0.24	9.10
Gini Index	0.3535	0.5278	0.3077	0.3662	0.5764	0.5072	0.504	0.3523	0.4276	-

Source: Data from the Ministry of Public Health.2011

**Table 2:** Linear regression for under 5 years infant and maternal mortality, and measles immunization coverage by health personnel density

Independent variables	Under 5 years infant mortality			Maternal mortality			Measles immunization coverage		
	Coefficient	t	*p*>/t/	Coefficient	t	*p*>/t/	Coefficient	t	*p*>/t/
Density of physicians	-9.622	-1.680	0.131	-3.036	-0.606	0.561	-12.844	-5.404	0.001
Density of Nurses	-1.252	-0.986	0.353	-1.307	-0.897	0.396	-1.751	-1.962	0.085
Density of paramedics	-4.926	-1.154	0.282	-3.941	-0.781	0.457	-8.128	-3.436	0.009
Combined density of Physicians, nurses and paramedics	-1.052	-1.206	0.262	-0.919	-0.895	0.397	-1.507	-2.738	0.026
R^2^	0.226			0.074			0.548		

*P>/t/=*P*-value

Source: Data from the Ministry of Public Health.2011

**Fig. 2:**
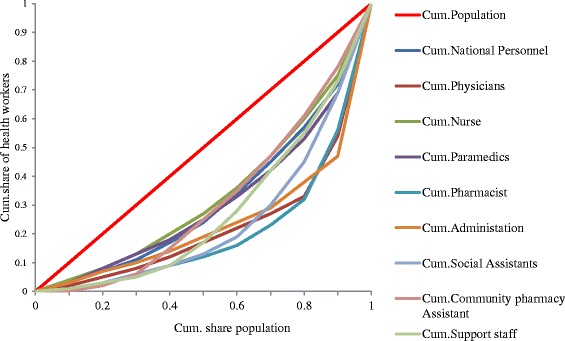
Lorenz curve for the distribution of all health personnel by cadre per region. 2011 *Cum. =Cumulative share Source: Data from the Ministry of Public Health.2011

Based on the regional population and the available health personnel, the overall inter-regional inequalities has a Gini coefficient of 0.3535 for nationwide health personnel, 0.5278 for physicians, 0.3077 for nurses, 0.3662 for paramedics, 0.5764 for pharmacist,0.5072 for Administrators,0.504 for Social assistant agents, 0.3523 for community pharmacist agents and 0.4276 for support staff. Table 1 and Fig. 2 shows the Gini index and the Lorenz curves for the distribution of health personnel per cadre respectively.

**Fig. 3:**
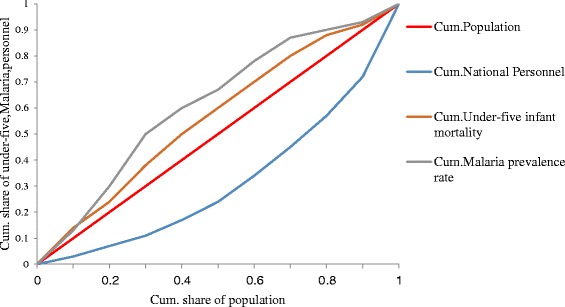
Distribution of national health personnel, malaria prevalence rate and under-five infant mortality. *Cum. =Cumulative share. Source: Data from the Ministry of Public Health.2011

Figure 3, shows the use of concentration curves superimposed on the same diagram with Lorenz curve for cumulative share of all health personnel, to illustrate the use of alternative indicators (cumulative share of under-five infant mortality and malaria prevalence rate) to population levels as a measure of health care needs. The concentration curves for under-five infant mortality and malaria prevalence rate lies above the diagonal, implying that regions with fewer number of available health personnel also shows a higher share of under-five mortality and malaria prevalence with concentration index of −0.1726 and −0.2871 respectively.

### Distribution of health personnel

**Fig. 4:**
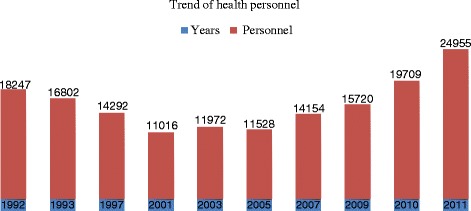
The number of public health personnel from 1992-2011. Source: Data from the Ministry of Public Health.2011. *Top figures represent total staff per the below years

Data on Table 3 revealed that, 24,955 (65.32 %) health personnel worked with the public health sector, and were deployed within the ten geographical regions of the country. The nurse cadre 13,081 (52.42 %) was the most represented category and the pharmacist cadre 55 (0.22 %) the least represented. The physicians with a 5.74 % of the national workforce had a percentage representation of <1 % in eight out of the ten regions of the nation. Data on Table 4 also revealed that, females occupy more than half of the national public sector health workforce with 13,909 (55.74 %) personnel, and 11,046 (44.26 %) personnel for males, leading to a male–female ratio of 1:1.26. The nurse cadre which is the most represented category had 8,169 (62.45 %) females and 4,912 (37.55 %) males, representing a male–female ratio of 1:1.26. Also, there were 9,139 (36.62 %) of health personnel within 31–40 years age group, and 4,779 (19.15 %) of age ≤30 years. All staff ages range from 20 to 66 years, with an overall 13,918 (55.8 %) of the health workforce falling within ≤40 years of age with mean (SD) of 36.4 $\pm 6$. We also observed a decline of 7,231 (60 %) of public health workforce from 1992–2001 and from 2003–2011, the sector gained 12,983 (52 %) personnel, Fig. 4.

**Table 3:** General population and percentage (%), of different cadre of health personnel and their geographic distribution per region 2011

Occupation regions	General population	General public health personnel	Physicians	Nurses	Paramedics	Pharmacist	Administration	Social assistant agents	Community pharmacist agents	Support staff
Adamawa	1,015,622 (5.23)	857 (3.43)	38 (0.15)	596 (2.39)	117 (0.47)	1 (0.00)	46 (0.18)	3 (0.01)	2 (0.01)	54 (0.22)
Centre	3,525,664 (18.17)	6930 (27.77)	647 (2.59)	3236 (13.00)	966 (3.87)	24 (0.10)	653 (2.62)	71 (0.28)	83 (0.33)	1250 (5.01)
East	801,968 (4.13)	1083 (4.34)	47 (0.19)	682 (2.73)	161 (0.65)	2 (0.01)	43 (0.19)	28 (0.11)	32 (0.13)	88 (0.35)
Extreme north	3,480,414 (17.93)	2715 (10.88)	70 (0.28)	1399 (5.61)	283 (1.13)	2 (0.01)	47 (0.19)	137 (0.55)	154 (0.62)	623 (2.50)
Littoral	2,865,795 (14.77)	3701 (14.83)	297 (1.19)	1889 (7.57)	477 (1.91)	5 (0.02)	108 (0.43)	11 (0.04)	108 (0.43)	806 (3.23)
North	1,050,229 (10.56)	1435 (5.75)	42 (0.17)	901 (3.61)	151 (0.61)	2 (0.01)	48 (0.19)	14 (0.06)	89 (0.36)	188 (0.75)
North West	1,804,695 (9.30)	1890 (7.57)	73 (0.29)	900 (3.61)	223 (0.89)	1 (0.00)	115 (0.46)	47 (0.19)	127 (0.51)	404 (1.62)
West	1,785,285 (9.20)	3066 (12.29)	91 (0.36)	1728 (6.92)	366 (1.47)	4 (0.02)	67 (0.27)	107 (0.43)	190 (0.76)	513 (2.06)
South	692,142 (3.57)	938 (3.76)	52 (0.21)	551 (2.21)	142 (0.57)	1 (0.00)	59 (0.24)	16 (0.06)	22 (0.09)	95 (0.38)
South West	1,384,286 (7.13)	2340 (9.38)	75 (0.30)	1199 (4.80)	239 (1.01)	13 (0.05)	64 (0.26)	16 (0.06)	93 (0.37)	641 (2.57)
Total	19,406,100 (100.00)	24955 (100.00)	1432 (5.74)	13081 (52.42)	3125 (12.52)	55 (0.22)	1250 (5.03)	450 (1.80)	900 (3.61)	4662 (18.68)

Source: Data from the Ministry of Public Health.2011

**Table 4:** Age and sex distribution of public health personnel by cadre. 2011

Age group (years)	10-20		21-30		31-40		41-50		51-60		61-70		Total cadre (%)	
F&(M	F	M	F	M	F	M	F	M	F	M	F	M	F	M
Physicians	0	0	162 (0.64)	133 (0.53)	211 (0.85)	312 (1.25)	123 (0.49)	296 (1.19)	52 (0.21)	118 (0.47)	6 (0.02)	18 (0.36)	554 (2.23)	878 (3.52)
Nurses	35 (0.14)	14 (0.06)	1628 (6.52)	705 (2.83)	3289 (13.18)	1523 (6.10)	2662 (10.67)	1870 (7.49)	545 (2.18)	784 (3.14)	10 (0.04)	16 (0.06)	8169 (32.73)	4912 (19.68)
Paramedics	4 (0.02)	7 (0.3)	335 (1.34)	245 (0.98)	608 (2.44)	478 (1.92)	426 (1.71)	580 (2.32)	153 (0.61)	285 (1.14)	1 (0.00)	3 (0.01)	1527 (6.12)	1598 (6.40)
Pharmacist	0	0 (0.00)	5 (0.02)	1 (0.00)	11 (0.04)	3 (0.01)	13 (0.05)	14 (0.06)	3 (0.01)	5 (0.02)	0 (0.00)	0 (0.00)	32 (0.13)	23 (0.09)
Administra-tion	3 (0.01)	1 (.0.01)	134 (0.54)	152 (0.61)	234 (0.94)	327 (1.31)	119 (0.48)	161 (0.65)	34 (0.14)	81 (0.34)	0 (0.00)	4 (0.02)	524 (2.10)	726 (2.91)
Social Assistant agents	7 (0.03)	4 (0.02)	42 (0.17)	48 (0.91)	117 (0.47)	81 (0.32)	58 (0.23)	45 (0.18)	15 (0.06)	18 (0.07)	2 (0.01)	13 (0.05)	241 (1.01)	209 (0.84)
Community pharmacist	12 (0.05)	5 (0.02)	131 (0.53)	58 (0.23)	222 (0.89)	161 (0.65)	147 (0.59)	106 (0.42)	35 (0.14)	16 (0.06)	2 (0.01)	5 (0.02)	549 (2.20)	351 (1.43)
support staff	29 (0.12)	24 (0.10)	493 (1.98)	362 (1.45)	800 (3.21)	762 (3.05)	731 (2.93)	731 (2.93)	245 (0.98)	337 (1.35)	15 (0.06)	135 (0.54)	2313 (9.27)	2349 (9.41)
Total (%)	90 (0.36)	55 (0.22)	2930 (11.74)	1704 (6.82)	5492 (22.00)	3647 (14.61)	4279 (17.15)	3802 (15.24)	1082 (4.34)	1644 (6.59)	36 (0.14)	194 (0.78)	13909 (55.74)	11046 (44.26)

*F=Female; M=Male

Source: Data from the Ministry of Public Health.2011

### Infrastructure and health personnel challenges

**Fig. 5:**
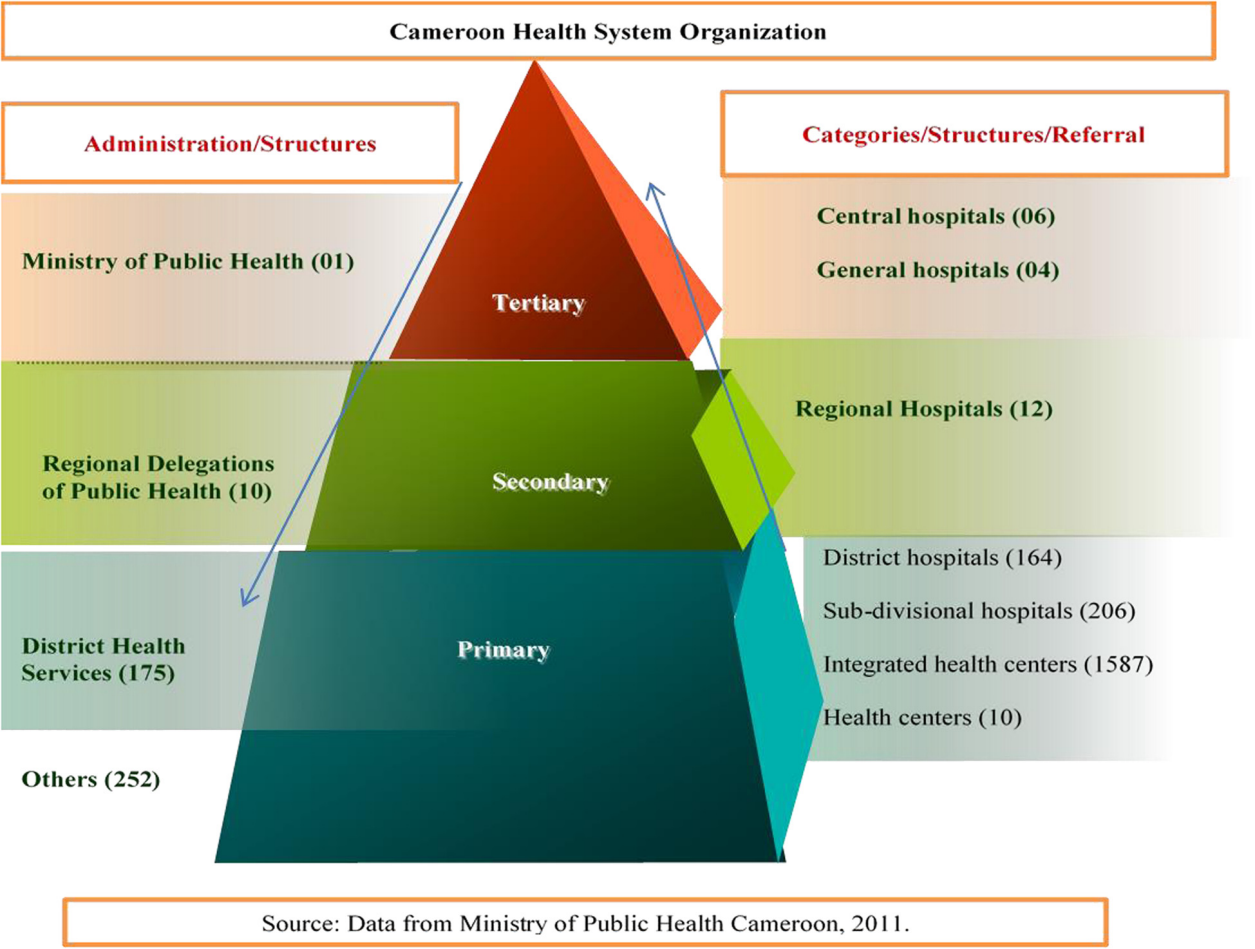
The Organization of the public health sector in Cameroon. 2011

**Fig. 6:**
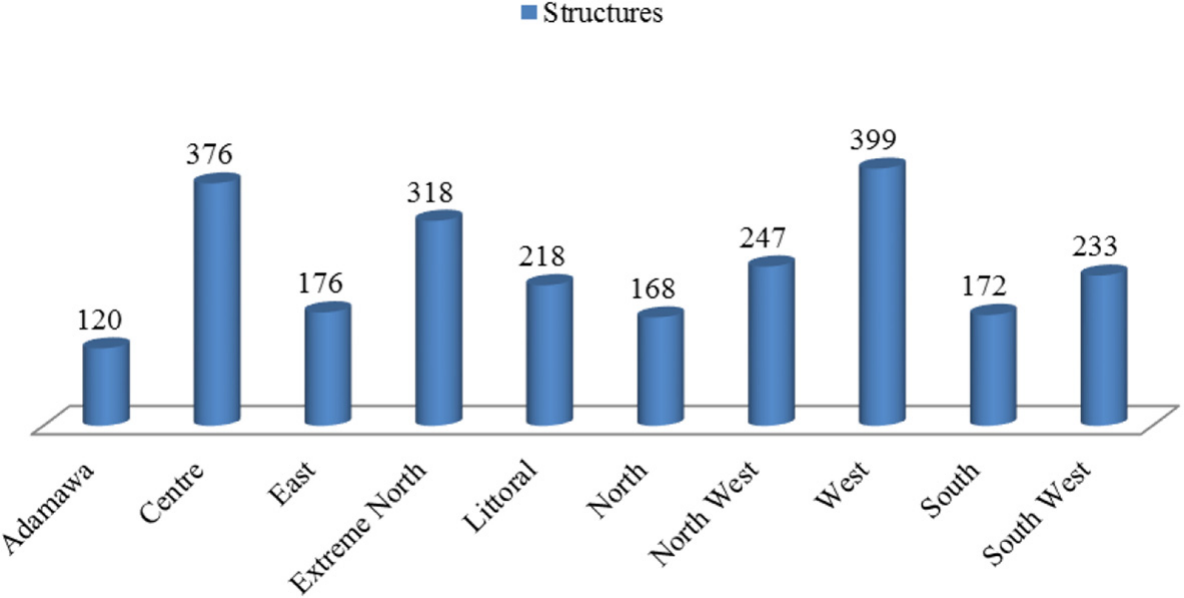
The geographical distribution of health structures across the regions.2011. *Figures represent the number of public health facilities per region. Source: Data from the Ministry of Public Health.2011

Of the 4,351 health facilities in Cameroon, 2,428 (55.80) are distributed within the public health sector of the nation. 2,175 (89.58 %) are directly involved with healthcare delivery services, 1,793 (73.85 %) are located at the primary level of health care services, 382 (15.73 %) at the secondary and tertiary levels, and 253 (10.42 %) involved with training and other services related to health as shown on Fig. 5. There was only one training school for physicians and 40 training centers for other health cadres distributed within the country, of which the Centre region had the highest (10) centers, while the North and South regions having only one center each [11]. The West 399 (16.49 %), the Centre 376 (15.49 %) and the Littoral 318 (13.10 %) regions were the most represented with health facilities, while the North 168 (6.92 %) and Adamawa 120 (4.94 %) regions were the least. Figure 6 shows the distribution of public health structures per region of the nation.

## Discussion

This study is a first attempt to describe and measure systematically the level of availability and distribution of the health workforce in Cameroon, using Lorenz curve and Gini coefficient as well as using linear regression analysis to measure health personnel density and selected health outcomes. It is equally the first attempt to use alternative indicators to surrogate population levels to determine health care needs when measuring health personnel distributional inequalities in Cameroon.

Our study results depicts that there is an overall inadequacy of the current stock of health personnel availability and significant inequalities in their distribution within the different regions in 2011.

The observed four-fold variation in densities of the workforce and the geographical trends in shortages and the uneven distribution of health personnel saw a departure in the provision of sufficient, fair and quality health-care services to the population. This may have accounted for the 95 deaths per 1,000 live births of neonates and the 36 % of malaria prevalence of the nation [24]. However, poor performing regions in terms of health outcomes have a major setback in the number of health workforce. For example, in the northern regions which are amongst the least represented with health personnel, we observed an estimated 61 % of unattended births and ≥35 % of diarrheal deaths among children [6, 11]. Some studies have shown the association between health personnel density to population as an important health system indicator in cross-country analyses [27, 28]. These shortages highlight the need to improve, develop and implement a sound, evidence-based health workforce plans targeting rural-poor and vulnerable regions and population.

The economic reforms imposed by the IMF and the World Bank saw the shrinkage in the public health sector recruitment and the development of other health resources and may have accounted for the shortage and distributional inequalities of the health workforce as well as led to the proliferation of the private sector [11]. These findings are further supported by similar studies performed in developing countries [29, 30]. Also, the unsuitable living and working environments, including salary cuts, among other factors prompted many public health personnel in Cameroon to either intend to migrate or migrated to other sectors or abroad for better conditions [30, 31].

In Cameroon, the centralized system of recruitment and deployment of health personnel has been termed “bureaucratic” as the period set from recruitment to a complete integration of a staff to reception of first salary could last for 36 months or more, while the personnel is expected to be at work during this period, a situation said to be complex and confusing [23]. Published literature shows that public health workers expressed dissatisfaction with working and living conditions due to low salary, overwork burden, no professional autonomy, limited opportunities for career progress and lack of sufficient as well as modern equipment for smooth and effective practices in Cameroon [6, 7, 19, 22].

Cameroon is in the course of devolution of its government as “regional balance” is currently practice in the recruitment of students into the few medical training schools according to their regions of origin across the nation [32] as a means to resolve issues surrounding cultural, ethnic and religious differences. This has been supported by similar studies performed on decentralization and health outcomes [33, 34], as one of the measures to effectively addressed HRH crisis.

In accordance with global trends, sex distribution of the available workforce shows a preponderance of females 13,909 (55.74 %) over the males 11,046 (44.26 %) leading to a male–female ratio of 1:1.2. This is in line with the WHO values of ≥70 % females as against ≤60 % for males of the health workforce worldwide [1]. Also the nurse cadre which is the most represented category of the workforce showed a male–female ratio of 1:2, as 8,169 (62.45 %) were female and 4,912 (37.55 %) males across the nation. The gender inequality that favors females is of prime importance in addressing staff retention and distribution. Thus the government may wish to consider gender balance during recruitment and deployment of staff.

Also, our data shows that more than half of the health personnel 55.77 % are ≤40 years of age. This may have been due to the freeze in hiring of public health staff during the economic crisis period and the revitalization in recruitment from 2003 to 2011, giving room for the integration of younger workforce [11]. This implies a long-term stability of the available health personnel, as many personnel still have longer productive working years before the retirement age set in Cameroon between 51 years to 60 years depending on staff qualification.

The occurrence of different disease profiles and epidemiological patterns across the geographical regions makes it unsuitable to analyse the distribution of health personnel according to need using the standard measure of population levels. In this study we proposed and show the use of alternative indicators (under-five infant mortality and malaria prevalence rate) as a surrogate to population levels to measure healthcare needs. By illustrating the indicators using concentration curves on the same diagram with Lorenz curve, we were able to show the actual and overall distribution of the national health workforce. Our alternative indicators shows a different representation from the standard measure of health need, but this deviation with respect to inequality may differ when different indicators are applied. Although malaria prevalence rate is higher than under-five mortality, both show higher concentrated in regions with proportional smaller share of health personnel. Also, by using the standard measure of population levels in determining distributional inequalities of the health workforce, much vital information regarding health care needs may be omitted. Although we have suggested the use of some alternative measures in this paper, there is the need for a more harmonise and comprehensive measures of health care needs that will cover the differences in disease profile and epidemiologic patterns across the different regions of the nation.

### Limitations

Our study presented the availability and distribution of public sector health workforce across the different regions of Cameroon. However, there are some limitations to our findings. There was a dearth of information regarding HRH as very little has been documented on this area in the country. Although, the data from the MoPH database is the most reliable in terms of quantity and quality of information, they may have been bias since the census was the first ever conducted on HRH in the country. The data informed on the general public health workforce and regional location but didn’t detail the specific health facility of staff and their specific professional qualifications. This makes it difficult to analyze what specific cadres were available in either primary, secondary or tertiary levels of the health system. Our work was limited to the public health sector workforce which is bias as when analyzing health systems, it is important to include all sectors that are involve in rendering health services to the population by including; the private, and not-for-profit non-governmental sectors. Also the use of only two alternative indicators for healthcare needs as a measure of distribution inequality of health personnel is a partial representation of a national healthcare needs as this requires multidisciplinary team work involving other ministries and professionals.

## Conclusion

This study revealed issues surrounding the inadequacy in the availability of health personnel in Cameroon, that is further complicated by the geographic distributional inequality and varying workforce characteristics (for example, sex and age profile) across the different regions. In addition to inequalities and the uneven distribution of health personnel, cultural, religious and ethnic differences also contributed in widening the degree of disease burden and health outcomes in some regions of the nation.

This study seeks for collaborative actions between the government of Cameroon, stakeholders, professionals, and international partners to direct policies that will address key issues surrounding HRH improvement. Such measures could involve facilitating recruitment, deployment, and integration processes of health personnel, provision of career progress opportunities (through scholarships and subsidies) and the construction and expansion of both training and health infrastructures across the nation. The training and retraining of personnel, including task shifting as many as possible from physicians, nurses, pharmacists and paramedics to non-clinical staff, giving room for clinicians to concentrate on complex and specific areas of expertise is of importance [35]. These are in line with the recommended WHO measures in increasing access of health personnel to underserved areas [36]. Furthermore, we urge other organizations other than the government to be involve in staff audits and facilities of the health system of Cameroon for a more specific and up-to-date information about the sector.

Moreover, health personnel retention does not depend solely on finances [21], but policy makers may consider creating a more conducive, competitive and attractive packages, and facilities that will encourage and retain those on the ground as well as return tickets for those studying and practicing abroad for their return to the country. The facilitation of the decentralization process will assist in the rapid identification, development and problem solving of specific regional and community’s need-base resources pertaining to health care needs as well as other sectors leading to the improvement of the societal wellbeing.

Also by combining concentration curves on the same diagram with Lorenz curves gives a visible illustration of the significance of using alternative indicators of health care needs to determine distributional inequalities of health personnel. However, population levels may be useful as an indicator to determine health personnel inequalities and health care needs in settings with uniform distribution of disease profile and epidemiologic pattern but as a bias in case where this varies across geographical area. Therefore depending solely on population levels as a measure of health care need may leave out vital information needed for a more reliable and effective distribution of health personnel according to health care needs.

Hence, there is the need for a collaborative multidisciplinary professional team work for the careful identification of indicators that will measure health care needs alongside policy implications for a proper resource planning and allocation.

## Abbreviations

HRH: Human resources for health
MDG: Millennium development goals
SAP: Structural adjustment program
IMF: International monetary fund
MoPH: Ministry of public health
NISC: National institute of statistics cameroon (NISC)
BUCREP: Central Bureau of census and population studies Cameroon
DMOs: District medical officers
SSA: Sub-Saharan Africa

## References

[1] World Health Organization (2006). The world health report: 2006: working together for health.

[2] Joint Learning Initiative (2004). Human resources for health: overcoming the crisis.

[3] Global health work force. Health workers for all and all for health workers: The human resource for health crisis, Geneva; 2013.

[4] Mubashar S. Trained health workers: A key to MDGs. Geneva, Switzerland. 2008.

[5] Dovlo D (2005). Wastage in the health workforce: some perspectives from African countries. Hum Resour Health.

[6] Amani A (2010). The health workers crises in Cameroon. Health.

[7] Fongwa Marie N (2002). International health care perspectives: the Cameroon example. J Transcult Nurs.

[8] Dovlo D (2007). Migration of nurses from Sub‐Saharan Africa: a review of issues and challenges. Health Serv Res.

[9] Hagopian A, Ofosu A, Fatusi A, Biritwum R, Essel A, Hart LG (2005). The flight of physicians from West Africa: views of African physicians and implications for policy. Soc Sci Med.

[10] USAID. Bureau for Africa, office of sustainable development. The Health Sector Human Resource Crisis in Africa: An Issues Paper. 2003.

[11] Ministry of Public Health Cameroon. Department of Human Resource. General census report of health personnel. Yaounde, 2012.

[12] Central Intelligence Agency. The World Fact book. USA; 2014. [https://www.cia.gov/library/publications/the-world-factbook/geos/cm.html]. (Accessed 12.14. 2014).

[13] BUCREP (2010). La population du Cameroun.

[14] Munga MA, Mæstad O (2009). Measuring inequalities in the distribution of health workers: the case of Tanzania. Hum Resour Health.

[15] Theodorakis PN, Mantzavinis GD, Rrumbullaku L, Lionis C, Trell E (2006). Measuring health inequalities in Albania: a focus on the distribution of general practitioners. Hum Resour Health.

[16] Gravelle H, Sutton M (2001). Inequality in the geographical distribution of general practitioners in England and Wales 1974-1995. J Health Serv Res Policy.

[17] Johnston G, Wilkinson D (2001). Increasingly inequitable distribution of general practitioners in Australia, 1986–96. Aust N Z J Public Health.

[18] National Institute for Statistics Cameroon. The national health profile. Yaounde; 2011.

[19] Policy brief on scaling up malaria control interventions in Cameroon. Centre for the development of best practices in health, WHO, Geneva .2010. [http://www.cdbph.org/]. Accessed 23.03.2014).

[20] Antonio-Nkondjio C, Defo-Talom B, Tagne-Fotso R, Tene-Fossog B, Ndo C, Lehman LG (2012). High mosquito burden and malaria transmission in a district of the city of Douala, Cameroon. BMC Infect Dis.

[21] Bernhard L (2004). The state of the health workforce in Sub-Saharan Africa: evidence of crisis and analysis of contributing factors. Africa Region Human Development Working Paper Series.

[22] Ngufor G (1999). Public service reforms and their impact on health sector personnel in Cameroon. Impact of public service reforms on health personnel.

[23] Ministry of Public Health Cameroon. National policies on human resources for health, Yaounde, Cameroon. 2011

[24] Pacqué-Margolis S, Ng C, Kauffman S. IntraHealth International Human Resources for Health (HRH) Indicator Compendium, Capacity Plus, USAID, Washington. 2011. [http://www.capacityplus.org/files/resources/HRH_Indicator_Compendium.pdf] (Accessed 12.2. 2014).

[25] Wagstaff A (2000). Socioeconomic inequalities in child mortality: comparisons across nine developing countries. Bull World Health Organ.

[26] Wagstaff A, Naoke W (1999). Socioeconomic inequalities in child malnutrition in the developing world. World Bank Policy Res Working Paper.

[27] Anand S, Bärnighausen T (2004). Human resources and health outcomes: cross-country econometric study. Lancet.

[28] Anand S, Bärnighausen T (2007). Health workers and vaccination coverage in developing countries: an econometric analysis. Lancet.

[29] Awases M, Gbary A, Nyoni J, Chatora R (2004). Migration of health professionals in six countries: a synthesis report. World Health Organization.

[30] Abena Obama MTS, Nko’o A, Afane Ze E (2003). La migration du personnel de la santé qualifié dans la region Afrique: Le cas du Cameroun. Health Sci Dis.

[31] McCourt W, Awases M (2007). Addressing the human resources crisis: *a case study of the Namibian health service*. Hum Resour Health.

[32] Ministry of Public Health Cameroon, 2010. Stratégie de Coopération de L’oms avec les Pays 2010-2015. WHO, Regional Office for Africa. 2010, 12: 5-13

[33] Lodenstein E, Dao D (2011). Devolution and human resources in primary healthcare in rural Mali. Hum Resour Health.

[34] Teklehaimanot HD, Teklehaimanot A (2013). Human resource development for a community-based health extension program: a case study from Ethiopia. Hum Resour Health.

[35] Ferrinho P, Siziya S, Goma F, Dussault G (2011). The human resource for health situation in Zambia: deficit and maldistribution. Hum Resour Health.

[36] World Health Organization (2010). Increasing access to health workers in remote and rural areas through improved retention: global policy recommendations.

